# Rényi Entropy Power Inequalities via Normal Transport and Rotation

**DOI:** 10.3390/e20090641

**Published:** 2018-08-26

**Authors:** Olivier Rioul

**Affiliations:** 1 LTCI, Télécom ParisTech, Université Paris-Saclay, 75013 Paris, France; olivier.rioul@telecom-paristech.fr or; 2 École Polytechnique, Université Paris-Saclay, 91128 Palaiseau, France

**Keywords:** Rényi entropy, entropy power inequalities, transportation arguments, normal distributions, escort distributions, log-concave distributions

## Abstract

Following a recent proof of Shannon’s entropy power inequality (EPI), a comprehensive framework for deriving various EPIs for the Rényi entropy is presented that uses transport arguments from normal densities and a change of variable by rotation. Simple arguments are given to recover the previously known Rényi EPIs and derive new ones, by unifying a multiplicative form with constant *c* and a modification with exponent α of previous works. In particular, for log-concave densities, we obtain a simple transportation proof of a sharp varentropy bound.

## 1. Introduction

The entropy power inequality (EPI) dates back to Shannon’s seminal paper [1] and has a long history [2]. The link with the Rényi entropy was first made by Dembo, Cover and Thomas [3] in connection with Young’s convolutional inequality with sharp constants, where Shannon’s EPI is obtained by letting the Rényi entropy orders tend to one ([4] Theorem 17.8.3).

The Rényi entropy [5] was first defined as a generalization of Shannon’s entropy for *discrete* variables, when looking for the most general definition of information measures that would preserve the additivity for independent events. It has found many applications such as source coding [6], hypothesis testing [7], channel coding [8] and guessing [9]. The (differential) Rényi entropy considered in this paper (Definition 2) generalizes the (differential) Shannon’s entropy for continuous variables. It was first considered in [3] to make the transition between the entropy-power and the Brunn–Minkowski inequalities. It has also been applied to deconvolution problems [10]. A definition of the Renyi entropy-power itself appears in [11], which is essentially Definition 5 below.

Recently, there has been significant interest in Rényi entropy power inequalities for several independent variables (the survey [12] is recommended to the reader for recent developments on forward and reverse EPIs). Bobkov and Chistyakov [13] extended the classical Shannon’s EPI to the Rényi entropy by incorporating a multiplicative constant that depends on the order of the Rényi entropy. Ram and Sason [14] improved the value of the constant by making it depend also on the number of variables. Bobkov and Marsiglietti [15] proved another modification of the EPI for the Rényi entropy for two independent variables, with a power exponent parameter α whose value was further improved by Li [16]. All these EPIs were found for Rényi entropies of orders >1. The α-modification of the Rényi EPI was extended to orders <1 for two independent variables having log-concave densities by Marsiglietti and Melbourne [17]. The starting point of all the above works was Young’s strengthened convolutional inequality.

Recently, Shannon’s original EPI was given a simple proof [18] using a transport argument from normal variables and a change of variable by rotation. In this paper, we exploit these ingredients, described in the following lemmas, to establish all the above-mentioned Rényi EPIs and derive new ones.

**Notation** **1.** Throughout this article, the considered n-dimensional zero-mean random variables $X\in R^{n}$ admit a density which is implicitly assumed continuous inside its support. We write X∼f if X has density f and write $X^{*}∼N\left(0,K\right)$ if $X^{*}$ is normally distributed with n×n covariance matrix K.

**Lemma** **1** (Normal Transport). Let $X^{*}∼N\left(0,\sigma^{2}I\right)$. There exists a diffeomorphism $T:R^{n}\to R^{n}$ such that $X=T(X^{*})∼f$. Moreover, T can be chosen such that its Jacobian matrix $T^{\prime }$ is (lower) triangular with positive diagonal elements.

Lemma 1 is known in optimal transport theory as an application of the Knothe–Rosenblatt map [19,20]. Two different proofs are given in [18]. The proof is very simple for one-dimensional variables [21], where *T* is just an increasing function with continuous derivative $T^{\prime }>0$.

**Lemma** **2** (Normal Rotation [18]). If $X^{*},Y^{*}$ are i.i.d. (independent and identically distributed) and normal, then for any 0<λ<1, the rotation

(1) X˜=λX*+1−λY*Y˜=−1−λX*+1λY*

*yields i.i.d. normal variables $\tilde{X},\tilde{Y}$.*

Notice that the starred variables can be expressed in terms of the tilde variables by the inverse rotation

(2) X*=λX˜−1−λY˜Y*=1−λX˜+1λY˜.

The proof of Lemma 2 is trivial considering covariance matrices. A deeper result states that this property of remaining i.i.d. by rotation characterizes the normal distribution—this is known as Bernstein’s lemma (see, e.g., ([21] [Lemma 4]) and ([22] [Chapter 5])). This explains why one obtains equality in the EPI only for normal variables (see [21] for more details).

This article is a revised, full version of what was presented in part in a previous conference communication [23]. It is organized as follows. Preliminary definitions and known properties are presented in Section 2. Section 3 derives a crucial “information inequality” for Rényi entropies that enjoys a transformational invariance. The central result is in Section 4, where the first version of the Rényi EPI by Dembo, Cover and Thomas is proven using the ingredients of Lemmas 1 and 2. All previously known Rényi EPIs for finite orders—and new ones—are then derived using a simple method in Section 5. Section 6 concludes.

## 2. Preliminary Definitions and Properties

Throughout this article, we consider exponents p>0 with p≠1. The following definition is well known and used, e.g., in Hölder’s inequality.

**Definition** **1** (Conjugate Exponent). *The conjugate exponent of p is*

(3) $$p^{\prime }=\frac{p}{p-1},$$

*that is, the number $p^{\prime }$ such that*

(4) $$\frac{1}{p}+\frac{1}{p^{\prime }}=1.$$

**Remark** **1.** *There are two situations depending on whether $p^{\prime }$ is positive or negative, as summarized in the following table:*

p>1
or
0<p<1

$p^{\prime }>1$

$p^{\prime }<0$

**Definition** **2** (Rényi Entropy). *If X has density $f\in L^{p}\left(R^{n}\right)$, its Rényi entropy of order p is defined by*

(5) $$h_{p}\left(X\right)=\frac{1}{1-p}\log\int_{R^{n}}f^{p}\left(x\right)\;dx=-p^{\prime }\log{∥f∥}_{p}$$

*where ${∥f∥}_{p}$ denotes the $L^{p}$ norm of f.*

It is known that the limit as p→1 is the Shannon entropy

(6) $$h_{1}\left(X\right)=-\int_{R^{n}}f\left(x\right)\log f\left(x\right)\;dx.$$

The Rényi entropy enjoys well-known properties similar to those of the Shannon entropy, which are recalled here for completeness.

**Lemma** **3** (Scaling Property). *For any a≠0,*

(7) $$h_{p}\left(aX\right)=h_{p}\left(X\right)+n\log\left|a\right|.$$

**Proof.**  Making a change of variables, $h_{p}\left(aX\right)=\frac{1}{1-p}\log\int {\left(\frac{1}{{\left|a\right|}^{n}}f\left(\frac{x}{a}\right)\right)}^{p}\;dx=\frac{1}{1-p}\log\int f^{p}\left(\frac{x}{a}\right)\frac{\;dx}{{\left|a\right|}^{n}}+\frac{1}{1-p}{\log|a|}^{n(1-p)}=h_{p}\left(X\right)+n\log\left|a\right|$. ☐

One recovers the usual scaling property for the Shannon entropy by letting p→1.

**Lemma** **4** (Rényi Entropy of the Normal). If $X^{*}∼N\left(0,K\right)$ for some nonsingular covariance matrix K, then

(8) $$h_{p}\left(X^{*}\right)=\frac{1}{2}\log({\left(2\pi \right)}^{n}\left|K|\right)+\frac{n}{2}p^{\prime }\frac{\log p}{p}$$

where |·| denotes the determinant. In particular, for $X^{*}∼N\left(0,\sigma^{2}I\right)$,

(9) $$h_{p}\left(X^{*}\right)=\frac{n}{2}\log\left(2\pi \sigma^{2}\right)+\frac{n}{2}p^{\prime }\frac{\log p}{p}.$$

**Proof.**  By direct calculation, $h_{p}\left(X^{*}\right)=\frac{1}{1-p}\log\int {\left(\frac{\exp(-\frac{1}{2}x^{t}K^{-1}x)}{\sqrt{{\left(2\pi \right)}^{n}\left|K\right|}}\right)}^{p}\;dx=\frac{1}{1-p}\log\frac{\sqrt{{\left(2\pi \right)}^{n}\left|K\right|p^{-n}}}{{\sqrt{{\left(2\pi \right)}^{n}\left|K\right|}}^{p}}=\frac{1}{2}\log({\left(2\pi \right)}^{n}\left|K|\right)-\frac{n}{2}\frac{\log p}{1-p}$. ☐

Again, one recovers the Shannon entropy of a normal variable by letting p→1 (then, $p^{\prime }\frac{\log p}{p}\to \log e$).

The following notion of escort distribution [24,25] is useful in the sequel.

**Definition** **3** (Escort Density ([25] § 2.2)). *If $f\in L^{p}\left(R^{n}\right)$, its escort density of exponent p is the density defined by*

(10) $$f_{p}\left(x\right)=\frac{f^{p}\left(x\right)}{\int_{R^{n}}f^{p}\left(x\right)\;dx}.$$

*In other words, $f_{p}=f^{p}/{∥f∥}_{p}^{p}$, where ${∥f∥}_{p}$ denotes the $L^{p}$ norm of f. We also use the notation $X_{p}$ to denote the corresponding escort random variable with density $f_{p}$.*

**Lemma** **5** (Monotonicity Property). *If p<q, then $h_{p}\left(X\right)\geq h_{q}\left(X\right)$ with equality if and only if X is uniformly distributed.*

**Proof.**  Let p≠1 and assume that $f\in L^{q}\left(R^{n}\right)$ for all *q* in a neighborhood of *p* so that one can freely differentiate under the integral sign:

(11) $$\begin{array}{cc} \frac{\partial }{\partial p}h_{p}\left(X\right) & =\frac{1}{{\left(1-p\right)}^{2}}\log\int f^{p}+\frac{1}{1-p}\frac{\int f^{p}\log f}{\int f^{p}}\\ & =\frac{1}{{\left(1-p\right)}^{2}}\left(\log\int f^{p}+\int f_{p}\log f^{1-p}\right)\\ & =\frac{1}{{\left(1-p\right)}^{2}}\int f_{p}\log\frac{f}{f_{p}}\\ & =-\frac{D(f_{p}∥f)}{{\left(1-p\right)}^{2}}\leq 0 \end{array}$$

where D(·∥·) denotes the Kullback–Leibler divergence. Equality $D(f_{p}∥f)=0$ can hold only if $f=f_{p}$ a.e., which, since p≠1, implies that *f* is constant over some measurable subset $A\subset R^{n}$, and is zero elsewhere. It follows that $h_{p}\left(X\right)>h_{q}\left(X\right)$ for any p<q if *X* is not uniformly distributed. Conversely, if *X* is uniformly distributed over some measurable subset $A\subset R^{n}$, its density can be written as f(x)=1/vol(A) for all x∈A and f(x)=0 elsewhere. Then, $h_{p}\left(X\right)=\frac{1}{1-p}\log\frac{\mathrm{vol}(A)}{\mathrm{vol}{\left(A\right)}^{p}}=\log\mathrm{vol}\left(A\right)$ is independent of *p*. ☐

**Remark** **2.** *Notice the identity established in the proof:*

(12) $$\frac{\partial }{\partial p}h_{p}\left(X\right)=-\frac{D(f_{p}∥f)}{{\left(1-p\right)}^{2}}.$$

*A similar formula for discrete variables can be found in ([26] § 5.3).*

## 3. An Information Inequality

The Shannon entropy satisfies a fundamental “information inequality” ([4] Theorem 2.6.3) from which many classical information-theoretic inequalities can be derived. This can be written as

(13) $$h_{1}\left(X\right)\leq -E\log\varphi \left(X\right)$$

for any density φ, with equality if and only if φ=f a.e. The following Theorem 1 can be seen as the natural extension of the information inequality to Rényi entropies and is central in the following derivations of this paper. Bercher [27] pointed out to the author that it is similar to an inequality for discrete distributions established by Campbell [6] in the context of source coding (see also [24]).

**Theorem** **1** (Information Inequality). *For any density φ,*

(14) $$h_{p}\left(X\right)\leq -p^{\prime }\log E\left(\varphi^{1/p^{\prime }}\left(X\right)\right)$$

*with equality if and only if $\varphi =f_{p}$ a.e.*

By letting p→1, one recovers the classical information inequality in Equation (13) for the Shannon entropy.

**Proof.**  By definition in Equation (5),

(15) $$\begin{array}{cc} h_{p}\left(X\right) & =-p^{\prime }\log{∥f∥}_{p}\\ & =-p^{\prime }\log{\left(\int (f^{p}\varphi^{-1})\varphi \right)}^{1/p}\\ & \leq -p^{\prime }\log\int {\left(f^{p}\varphi^{-1}\right)}^{1/p}\varphi \\ & =-p^{\prime }\log\int f\varphi^{1/p^{\prime }} \end{array}$$

where the inequality follows from Jensen’s inequality applied to the function $x\mapsto x^{1/p}$, which is strictly concave if p>1 (that is, $p^{\prime }>0$) and strictly convex if p<1 (that is, $p^{\prime }<0$). Equality holds if and only if $f^{p}\varphi^{-1}$ is constant a.e., which means that φ and $f^{p}$ are proportional a.e. Normalizing gives the announced condition $\varphi =f_{p}$ a.e. ☐

**Remark** **3.** *An alternate proof is obtained using Hölder’s inequality or its reverse applied to f and $\varphi^{1/p^{\prime }}$. Notice that the equality case for $\varphi =f_{p}$ gives*

(16) $$h_{p}\left(X\right)=-p^{\prime }\log E\left(f_{p}^{1/p^{\prime }}\left(X\right)\right)$$

*as can be easily checked directly.*

The following conditional version of Theorem 1 involves a more complicated relation for dependent variables.

**Corollary** **1** (Conditional Information Inequality) *For any two random variables $X,Y\in R^{n}$,*

(17) $$-p^{\prime }\log E_{Y}\exp\left(-h_{p}\left(X|Y\right)/p^{\prime }\right)\leq -p^{\prime }\log E\left(\varphi^{1/p^{\prime }}\left(X|Y\right)\right)$$

*where $h_{p}\left(X|y\right)$ denotes the Rényi entropy of X knowing Y=y and the expectation on the l.h.s. is taken over Y (the expectation in the r.h.s. is taken over (X,Y)).*

*In particular, when X and Y are independent,*

(18) $$h_{p}\left(X\right)\leq -p^{\prime }\log E\left(\varphi^{1/p^{\prime }}\left(X|Y\right)\right).$$

*with equality if and only if φ(x|y) does not depend on y and equals $f_{p}\left(x\right)$ a.e.*

**Proof.**  From Equation (14) for fixed *y*, one has $E\left(\varphi^{1/p^{\prime }}\left(X|y\right)\right)\leq \exp\left(-h_{p}\left(X|y\right)/p^{\prime }\right)$ for $p^{\prime }>0$ (p>1) and the opposite inequality for $p^{\prime }<0$ (p<1), with equality if and only if $\varphi \left(x|y\right)=f_{p}\left(x|y\right)$ a.e. Taking the expectation over *Y* yields $E\left(\varphi^{1/p^{\prime }}\left(X|Y\right)\right)\leq E_{Y}\exp\left(-h_{p}\left(X|Y\right)/p^{\prime }\right)$ for $p^{\prime }>0$ (p>1) and the opposite inequality for $p^{\prime }<0$ (p<1). The result follows by taking the logarithm and multiplying by $-p^{\prime }$. When *X* and *Y* are independent, equality holds if and only if $\varphi \left(x|y\right)=f_{p}\left(x\right)$ a.e. for all *y*. ☐

For the Shannon entropy, the difference between the two sides of the information inequality in Equation (13) is the Kullback–Leibler divergence:

$$D\left(f∥\varphi \right)=E\log\frac{f(X)}{\varphi (X)}\geq 0$$

which can also be noted D(X∥Z) where X∼f and Z∼φ. It is known (and easy to check) that the divergence is invariant by reversible transformations *T*. This means that, when $X=T(X^{*})$ and $Z=T(Z^{*})$, one has $D\left(X∥Z\right)=D\left(X^{*}∥Z^{*}\right)$. A natural extension to Rényi entropies can be obtained on the difference

(19) $$-p^{\prime }\log E\left(\varphi^{1/p^{\prime }}\left(X\right)\right)-h_{p}\left(X\right)\geq 0$$

between the two sides of the information inequality in Equation (14).

**Theorem** **2** (Transformational Invariance). *Let $T:R^{n}\to R^{n}$ be a diffeomorphism and suppose that*

(20) $$\begin{array}{cc} X_{p} & =T(X_{p}^{*}) \end{array}$$

(21) $$\begin{array}{cc} Z & =T(Z^{*}) \end{array}$$

where Z∼φ and $Z^{*}∼\varphi^{*}$. Then,

(22) $$-p^{\prime }\log E\left(\varphi^{1/p^{\prime }}\left(X\right)\right)-h_{p}\left(X\right)=-p^{\prime }\log E\left({\varphi^{*}}^{1/p^{\prime }}\left(X^{*}\right)\right)-h_{p}\left(X^{*}\right).$$

Note that, from Equation (5), this identity can be rewritten as

(23) $$\frac{E\left({\varphi^{*}}^{1/p^{\prime }}\left(X^{*}\right)\right)}{∥f^{*}{∥}_{p}}=\frac{E\left(\varphi^{1/p^{\prime }}\left(X\right)\right)}{{∥f∥}_{p}}.$$

**Proof.**  Proceed to prove Equation (23). Let $f,f^{*}$ be the respective densities of $X,X^{*}$ and recall that $X_{p}∼f_{p}$ and $X_{p}^{*}∼f_{p}^{*}$. By the transformation *T*, the densities are related by

(24) $$\begin{array}{cc} f_{p}^{*}\left(x^{*}\right) & =f_{p}\left(T\left(x^{*}\right)\right)\left|T^{\prime }\left(x^{*}\right)\right| \end{array}$$

(25) $$\begin{array}{cc} \varphi^{*}\left(x^{*}\right) & =\varphi \left(T\left(x^{*}\right)\right)\left|T^{\prime }\left(x^{*}\right)\right| \end{array}$$

where $|T^{\prime }|$ denotes the Jacobian determinant of *T*. Using these relations and Definition 3,

(26) $$\begin{array}{cc} E\left({\varphi^{*}}^{1/p^{\prime }}\left(X^{*}\right)\right)/{∥f^{*}∥}_{p} & =E\left(\varphi^{1/p^{\prime }}\left(T\left(X^{*}\right)\right){\left|T^{\prime }\left(X^{*}\right)\right|}^{1/p^{\prime }}\right)/{∥f^{*}∥}_{p}\\ & =\int \varphi^{1/p^{\prime }}\left(T\left(x^{*}\right)\right)|T^{\prime }\left(x^{*}\right){|}^{1/p^{\prime }}f^{*}\left(x^{*}\right)\;dx^{*}/{∥f^{*}∥}_{p}\\ & =\int \varphi^{1/p^{\prime }}\left(T\left(x^{*}\right)\right){\left|T^{\prime }\left(x^{*}\right)\right|}^{1/p^{\prime }}f_{p}^{*}{\left(x^{*}\right)}^{1/p}\;dx^{*}\\ & =\int \varphi^{1/p^{\prime }}\left(T\left(x^{*}\right)\right)f_{p}{\left(T\left(x^{*}\right)\right)}^{1/p}\left|T^{\prime }\left(x^{*}\right)\right|\;dx^{*}\\ & =\int \varphi^{1/p^{\prime }}\left(x\right)f_{p}{\left(x\right)}^{1/p}\;dx\\ & =E\left(\varphi^{1/p^{\prime }}\left(X\right)\right)/{∥f∥}_{p}.\;☐ \end{array}$$

**Remark** **4.** *φ is a density and is not used in the proof of Theorem 2. Therefore, Equation (22) holds more generally for any function φ satisfying Equation (25).*

## 4. First Version of the Rényi EPI

For two independent random variables *X* and *Y*, the Shannon entropy power inequality can be expressed as follows [2,3]: For any 0<λ<1,

(27) $$h\left(\sqrt{\lambda }X+\sqrt{1-\lambda }Y\right)\geq \lambda h\left(X\right)+\left(1-\lambda \right)h\left(Y\right)$$

with equality if and only if X,Y are i.i.d. normal—more precisely (given the translation invariance of the entropy) when they are normal with identical covariance matrices; since it was assumed in Notation 1 that all considered variables have zero mean, both statements are equivalent. This means that the difference $h\left(\sqrt{\lambda }X+\sqrt{1-\lambda }Y\right)-\lambda h\left(X\right)-\left(1-\lambda \right)h\left(Y\right)$ is minimum (zero) for i.i.d. normal X,Y. In this section, we study the natural generalization for Rényi entropies ([3] Theorem 12), namely that the quantity

(28) $$h_{r}\left(\sqrt{\lambda }X+\sqrt{1-\lambda }Y\right)-\lambda h_{p}\left(X\right)-\left(1-\lambda \right)h_{q}\left(Y\right)$$

is minimum for X,Y i.i.d. normal. Here, the triple (p,q,r) and its associated λ satisfy the following condition, which is used, e.g., in Young’s convolutional inequality.

**Definition** **4** (Exponent Triple). *An exponent triple ${\left(p,q,r\right)}_{\lambda }$ has conjugates $p^{\prime },q^{\prime },r^{\prime }$ of the same sign and such that*

(29) $$\frac{1}{p^{\prime }}+\frac{1}{q^{\prime }}=\frac{1}{r^{\prime }}.$$

*The corresponding coefficient λ∈(0,1) is defined by*

(30) $$\lambda =\frac{r^{\prime }}{p^{\prime }}=1-\frac{r^{\prime }}{q^{\prime }}$$

In other words, the exponents p,q,r are such that

(31) $$\frac{1}{p}+\frac{1}{q}=1+\frac{1}{r}$$

and fulfill one the following two conditions:
p,q,r>1or0<p,q,r<1$p^{\prime },q^{\prime },r^{\prime }>1$$p^{\prime },q^{\prime },r^{\prime }<0$$r^{\prime }<p^{\prime }$, $r^{\prime }<q^{\prime }$
$|r^{\prime }\left|<\right|p^{\prime }|$, $|r^{\prime }\left|<\right|q^{\prime }|$r>p, r>q
r<p, r<q

The key argument used in this section is the following. If X∼f and Y∼g, then for the escort variables, $X_{p}∼f_{p}$ and $Y_{q}∼g_{q}$. By normal transport (Lemma 1), one can write

(32) $$\begin{array}{cc} X_{p} & =T(X_{p}^{*}) \end{array}$$

(33) $$\begin{array}{cc} Y_{q} & =U(Y_{q}^{*}) \end{array}$$

for two diffeomorphims *T* and *U*, where $X^{*},Y^{*}$ are, say, i.i.d. standard normal N(0,I). (It follows that $X_{p}^{*}∼N\left(0,I/p\right)$ and $Y_{q}^{*}∼N\left(0,I/q\right)$.) We then have the following straightforward extension of Theorem 2.

**Lemma** **6** (Transformational Invariance for Two Independent Variables). *For a two-dimensional φ(x,y),*

(34) $$\begin{array}{cc} -r^{\prime }\log E\left(\varphi^{1/r^{\prime }}\left(X,Y\right)\right) & -\lambda h_{p}\left(X\right)-\left(1-\lambda \right)h_{q}\left(Y\right)\\ & =-r^{\prime }\log E\left({\varphi^{*}}^{1/r^{\prime }}\left(X^{*},Y^{*}\right)\right)-\lambda h_{p}\left(X^{*}\right)-\left(1-\lambda \right)h_{q}\left(Y^{*}\right) \end{array}$$

*where*

(35) $$\varphi^{*}\left(x^{*},y^{*}\right)=\varphi \left(T\left(x^{*}\right),U\left(y^{*}\right)\right)|T^{\prime }\left(x^{*}\right){|}^{\lambda }{\left|U^{\prime }\left(y^{*}\right)\right|}^{1-\lambda }.$$

**Proof.**  From Equation (5) and the definition of λ, Equation (34) can be rewritten as

(36) $$\frac{E\left({\varphi^{*}}^{1/r^{\prime }}\left(X^{*},Y^{*}\right)\right)}{∥f^{*}{∥}_{p}{∥g^{*}∥}_{q}}=\frac{E\left(\varphi^{1/r^{\prime }}\left(X,Y\right)\right)}{{∥f∥}_{p}{∥g∥}_{q}}.$$

By the transformations *T* and *U*, the densities of the escort variables are related by $f_{p}^{*}\left(x^{*}\right)\;=\;f_{p}\left(T\left(x^{*}\right)\right)\left|T^{\prime }\left(x^{*}\right)\right|$ and $g_{q}^{*}\left(y^{*}\right)=g_{q}\left(U\left(y^{*}\right)\right)\left|U^{\prime }\left(y^{*}\right)\right|$. Now, by the same calculation as in the proof of Theorem 2,

(37) $$\begin{array}{cc} \frac{E\left({\varphi^{*}}^{1/r^{\prime }}\left(X^{*},Y^{*}\right)\right)}{∥f^{*}{∥}_{p}{∥g^{*}∥}_{q}} & =\frac{E\left(\varphi^{1/r^{\prime }}\left(T\left(X^{*}\right),U\left(Y^{*}\right)\right)|T^{\prime }\left(X^{*}\right){|}^{1/p^{\prime }}{\left|U^{\prime }\left(Y^{*}\right)\right|}^{1/q^{\prime }}\right)}{∥f^{*}{∥}_{p}{∥g^{*}∥}_{q}}\\ & =\frac{\int \varphi^{1/r^{\prime }}\left(T\left(x^{*}\right),U\left(y^{*}\right)\right)|T^{\prime }\left(x^{*}\right){|}^{1/p^{\prime }}{\left|U^{\prime }\left(y^{*}\right)\right|}^{1/q^{\prime }}f^{*}\left(x^{*}\right)g^{*}\left(y^{*}\right)\;dx^{*}\;dy^{*}}{∥f^{*}{∥}_{p}{∥g^{*}∥}_{q}}\\ & =\int \varphi^{1/r^{\prime }}\left(T\left(x^{*}\right),U\left(y^{*}\right)\right)|T^{\prime }\left(x^{*}\right){|}^{1/p^{\prime }}{\left|U^{\prime }\left(y^{*}\right)\right|}^{1/q^{\prime }}f_{p}^{*}{\left(x^{*}\right)}^{1/p}g_{q}^{*}{\left(y^{*}\right)}^{1/q}\;dx^{*}\;dy^{*}\\ & =\int \varphi^{1/r^{\prime }}\left(T\left(x^{*}\right),U\left(y^{*}\right)\right)|T^{\prime }\left(x^{*}\right)\left|\right|U^{\prime }\left(y^{*}\right)|f_{p}{\left(T\left(x^{*}\right)\right)}^{1/p}g_{q}{\left(U\left(y^{*}\right)\right)}^{1/q}\;dx^{*}\;dy^{*}\\ & =\int \varphi^{1/r^{\prime }}\left(x,y\right)f_{p}{\left(x\right)}^{1/p}g_{q}{\left(y\right)}^{1/q}\;dx\;dy\\ & =\frac{E\left(\varphi^{1/r^{\prime }}\left(X,Y\right)\right)}{{∥f∥}_{p}{∥g∥}_{q}}.\;☐ \end{array}$$

**Lemma** **7.** *Let φ be the density of $\sqrt{\lambda }X+\sqrt{1-\lambda }Y$. Then,*

(38) $$\begin{array}{cc} h_{r}(\sqrt{\lambda }X & +\sqrt{1-\lambda }Y)-\lambda h_{p}\left(X\right)-\left(1-\lambda \right)h_{q}\left(Y\right)\\ & \geq -r^{\prime }\log E\left\{{\left(\varphi_{r}\left(\sqrt{\lambda }T\left(X^{*}\right)+\sqrt{1-\lambda }U\left(Y^{*}\right)\right)\cdot \left|\lambda T^{\prime }\left(X^{*}\right)+\left(1-\lambda \right)U^{\prime }\left(Y^{*}\right)\right|\right)}^{1/r^{\prime }}\right\}\\ & \;-\lambda h_{p}\left(X^{*}\right)-\left(1-\lambda \right)h_{q}\left(Y^{*}\right). \end{array}$$

**Proof.**  By the equality case of Theorem 1 (see (16)), one has

(39) $$h_{r}\left(\sqrt{\lambda }X+\sqrt{1-\lambda }Y\right)=-r^{\prime }\log E\varphi_{r}^{1/r^{\prime }}\left(\sqrt{\lambda }X+\sqrt{1-\lambda }Y\right).$$

Now, by Lemma 6 applied to $\varphi \left(x,y\right)=\varphi_{r}\left(\sqrt{\lambda }x+\sqrt{1-\lambda }y\right)$, we have $\varphi^{*}\left(x^{*},y^{*}\right)=\varphi_{r}\left(\sqrt{\lambda }T\left(x^{*}\right)+\sqrt{1-\lambda }U\left(y^{*}\right)\right)|T^{\prime }\left(x^{*}\right){|}^{\lambda }{\left|U^{\prime }\left(y^{*}\right)\right|}^{1-\lambda }$, and, therefore,

(40) $$\begin{array}{cc} h_{r}(\sqrt{\lambda }X & +\sqrt{1-\lambda }Y)-\lambda h_{p}\left(X\right)-\left(1-\lambda \right)h_{q}\left(Y\right)\\ & =-r^{\prime }\log E\left\{{\left(\varphi_{r}\left(\sqrt{\lambda }T\left(X^{*}\right)+\sqrt{1-\lambda }U\left(Y^{*}\right)\right)\cdot |T^{\prime }\left(X^{*}\right){|}^{\lambda }{\left|U^{\prime }\left(Y^{*}\right)\right|}^{1-\lambda }\right)}^{1/r^{\prime }}\right\}\\ & \;-\lambda h_{p}\left(X^{*}\right)-\left(1-\lambda \right)h_{q}\left(Y^{*}\right). \end{array}$$

Since from Lemma 1, *T* and *U* can be chosen such that $T^{\prime }$ and $U^{\prime }$ are (lower) triangular with positive diagonal elements, it follows easily from the arithmetic-geometric mean inequality that

(41) $$|T^{\prime }\left(X^{*}\right){|}^{\lambda }|U^{\prime }\left(Y^{*}\right){|}^{1-\lambda }\leq \left|\lambda T^{\prime }\left(X^{*}\right)+\left(1-\lambda \right)U^{\prime }\left(Y^{*}\right)\right|.$$

The result follows at once (for either positive or negative $r^{\prime }$). ☐

We can now use the normal rotation Lemma 2 to conclude by proving the following,

**Theorem** **3** (Rényi EPI [3]). *For independent X,Y and exponent triple ${\left(p,q,r\right)}_{\lambda }$,*

(42) $$h_{r}\left(\sqrt{\lambda }X+\sqrt{1-\lambda }Y\right)-\lambda h_{p}\left(X\right)-\left(1-\lambda \right)h_{q}\left(Y\right)\geq \frac{n}{2}r^{\prime }\left(\frac{\log r}{r}-\frac{\log p}{p}-\frac{\log q}{q}\right)$$

*with equality if and only if X,Y are i.i.d. normal.*

**Proof.**  If X,Y are i.i.d. normal, then $\sqrt{\lambda }X+\sqrt{1-\lambda }Y$ is also identically distributed as *X* and *Y*, and from Lemma 4, it is immediate to check that equality holds (irrespective of their covariances). Therefore, the inequality in Equation (42) is equivalent to

(43) $$h_{r}\left(\sqrt{\lambda }X+\sqrt{1-\lambda }Y\right)-\lambda h_{p}\left(X\right)-\left(1-\lambda \right)h_{q}\left(Y\right)\geq h_{r}\left(\sqrt{\lambda }X^{*}+\sqrt{1-\lambda }Y^{*}\right)-\lambda h_{p}\left(X^{*}\right)-\left(1-\lambda \right)h_{q}\left(Y^{*}\right)$$

where $X^{*}$, $Y^{*}$ are, say, i.i.d. standard normal N(0,I).

To prove Equation (43), consider the normal rotation of Lemma 2 and write $X^{*},Y^{*}$ in terms of $\tilde{X},\tilde{Y}$ using Equation (2) in the first term of the r.h.s. of Equation (38) (Lemma 7). One obtains:

(44) $$\begin{array}{cc} h_{r}(\sqrt{\lambda }X & +\sqrt{1-\lambda }Y)-\lambda h_{p}\left(X\right)-\left(1-\lambda \right)h_{q}\left(Y\right)\\ & \geq -r^{\prime }\log E\left\{{\left(\psi (\tilde{X}|\tilde{Y})\right)}^{1/r^{\prime }}\right\}-\lambda h_{p}\left(X^{*}\right)-\left(1-\lambda \right)h_{q}\left(Y^{*}\right), \end{array}$$

where

(45) $$\begin{array}{cc} \psi (\tilde{x}|\tilde{y})= & \varphi_{r}\left(\sqrt{\lambda }T\left(\sqrt{\lambda }\tilde{x}-\sqrt{1-\lambda }\tilde{y}\right)+\sqrt{1-\lambda }U\left(\sqrt{1-\lambda }\tilde{x}+\sqrt{\lambda }\tilde{y}\right)\right)\\ & \times |\lambda T^{\prime }\left(\sqrt{\lambda }\tilde{x}-\sqrt{1-\lambda }\tilde{y}\right)+\left(1-\lambda \right)U^{\prime }\left(\sqrt{1-\lambda }\tilde{x}+\sqrt{\lambda }\tilde{y}\right)|. \end{array}$$

Making the change of variable $z=\sqrt{\lambda }T\left(\sqrt{\lambda }\tilde{x}-\sqrt{1-\lambda }\tilde{y}\right)+\sqrt{1-\lambda }U\left(\sqrt{1-\lambda }\tilde{x}+\sqrt{\lambda }\tilde{y}\right)$, one obtains

(46) $$\int \psi \left(\tilde{x}|\tilde{y}\right)\;d\tilde{x}=\int \varphi_{r}\left(z\right)\;dz=1,$$

since $\varphi_{r}$ is a density. Hence, $\psi (\tilde{x}|\tilde{y})$ is also a density in $\tilde{x}$ for fixed $\tilde{y}$. Now, since by Lemma 2, $\tilde{X}$ and $\tilde{Y}$ are independent, by the conditional information inequality in Equation (18) of Corollary 1, one has

(47) $$-r^{\prime }\log E\left\{{\left(\psi (\tilde{X}|\tilde{Y})\right)}^{1/r^{\prime }}\right\}\geq h_{r}\left(\tilde{X}\right)=h_{r}\left(\sqrt{\lambda }X^{*}+\sqrt{1-\lambda }Y^{*}\right).$$

Combining with Equation (44) yields the announced inequality in Equation (43).

It remains to settle the equality case in Equation (43). From the above proof, equality holds in Equation (43) if and only if both Equation (41) and Equation (47) are equalities. Equality in Equation (41) holds if and only if for all i=1,2,…,n,

(48) $$\frac{\partial T_{i}}{\partial x_{i}}\left(X^{*}\right)=\frac{\partial U_{i}}{\partial y_{i}}\left(Y^{*}\right)\;a.s.$$

Since $X^{*}$ and $Y^{*}$ are independent normal variables, this implies that $\frac{\partial T}{\partial x_{i}}$ and $\frac{\partial U}{\partial y_{i}}$ are constant and equal. In particular the Jacobian $|\lambda T^{\prime }\left(\sqrt{\lambda }\tilde{x}-\sqrt{1-\lambda }\tilde{y}\right)+\left(1-\lambda \right)U^{\prime }\left(\sqrt{1-\lambda }\tilde{x}+\sqrt{\lambda }\tilde{y}\right)|$ in (45) is constant.

From Corollary 1 equality in Equation (47) holds if and only if $\psi (\tilde{x}|\tilde{y})$ does not depend on $\tilde{y}$, which implies that $\sqrt{\lambda }T\left(\sqrt{\lambda }\tilde{x}-\sqrt{1-\lambda }\tilde{y}\right)+\sqrt{1-\lambda }U\left(\sqrt{1-\lambda }\tilde{x}+\sqrt{\lambda }\tilde{y}\right)$ does not depend on the value of $\tilde{y}$. Taking derivatives with respect to $y_{j}$ for all j=1,2,…,n,

(49) $$-\sqrt{\lambda }\sqrt{1-\lambda }\frac{\partial T_{i}}{\partial x_{j}}\left(\sqrt{\lambda }\tilde{X}-\sqrt{1-\lambda }\tilde{Y}\right)+\sqrt{\lambda }\sqrt{1-\lambda }\frac{\partial U_{i}}{\partial x_{j}}\left(\sqrt{1-\lambda }\tilde{X}+\sqrt{\lambda }\tilde{Y}\right)=0$$

which implies

(50) $$\frac{\partial T_{i}}{\partial x_{j}}\left(X^{*}\right)=\frac{\partial U_{i}}{\partial y_{j}}\left(Y^{*}\right)\;a.s.$$

for all i,j=1,2,…,n. Therefore, *T* and *U* are linear transformations, equal up to an additive constant (equal to 0 since all variables are assumed of zero mean). It follows that $X_{p}=T\left(X_{p}^{*}\right)$ and $Y_{q}=U\left(Y_{q}^{*}\right)$ are normal with respective distributions $X_{p}∼N\left(0,K/p\right)$ and $Y_{q}∼N\left(0,K/q\right)$. Hence, *X* and *Y* are i.i.d. normal N(0,K). ☐

A straightforward generalization to several independent variables is the following.

**Corollary** **2** (Rényi EPI for Several Variables). *Let $r_{1},r_{2},\ldots ,r_{m},r$ be exponents; those conjugates $r_{1}^{\prime },r_{2}^{\prime },\ldots ,r_{m}^{\prime },r^{\prime }$ are of the same sign and satisfy*

(51) $$\sum_{i=1}^{m}\frac{1}{r_{i}^{\prime }}=\frac{1}{r^{\prime }}$$

*and let $\lambda_{1},\lambda_{2},\ldots ,\lambda_{m}$ be defined by*

(52) $$\lambda_{i}=\frac{r^{\prime }}{r_{i}^{\prime }}\;\left(i=1,2,\ldots ,m\right).$$

*Then, for independent $X_{1},X_{2},\ldots ,X_{m}$,*

(53) $$h_{r}\left(\sum_{i=1}^{m}\sqrt{\lambda_{i}}X_{i}\right)-\sum_{i=1}^{m}\lambda_{i}h_{r_{i}}\left(X_{i}\right)\geq \frac{n}{2}r^{\prime }\left(\frac{\log r}{r}-\sum_{i=1}^{m}\frac{\log r_{i}}{r_{i}}\right)$$

*with equality if and only if the $X_{i}$ are i.i.d. normal.*

**Proof.**  By induction on *m*: The result for m=2 is Theorem 3. Suppose the result satisfied at order m−1 and let $Y_{m}=\sum_{i=1}^{m-1}\sqrt{\lambda_{i}}X_{i}/\sqrt{1-\lambda_{m}}$ and $s_{m}$ be such that $\frac{1}{s_{m}^{\prime }}=\sum_{i=1}^{m-1}\frac{1}{r_{i}^{\prime }}$. Notice that $\frac{1}{r^{\prime }}=\frac{1}{r_{m}^{\prime }}+\frac{1}{s_{m}^{\prime }}=\frac{\lambda_{m}}{r^{\prime }}+\frac{1}{s_{m}^{\prime }}$, hence $r^{\prime }=\left(1-\lambda_{m}\right)s_{m}^{\prime }$. By Theorem 3, $h_{r}\left(\sum_{i=1}^{m}\sqrt{\lambda_{i}}X_{i}\right)=h_{r}\left(\sqrt{\lambda_{m}}X_{m}+\sqrt{1-\lambda_{m}}Y_{m}\right)\geq \lambda_{m}h_{r_{m}}\left(X_{m}\right)+\left(1-\lambda_{m}\right)h_{s_{m}}\left(Y_{m}\right)+\frac{n}{2}r^{\prime }\left(\frac{\log r}{r}-\frac{\log r_{m}}{r_{m}}-\frac{\log s_{m}}{s_{m}}\right)$ with equality if and only if $X_{m},Y_{m}$ are i.i.d. normal. Now, by the induction hypothesis, $h_{s_{m}}\left(Y_{m}\right)\geq \sum_{i=1}^{m-1}\frac{\lambda_{i}}{1-\lambda_{m}}h_{r_{i}}\left(X_{i}\right)+\frac{n}{2}s_{m}^{\prime }\left(\frac{\log s_{m}}{s_{m}}-\sum_{i=1}^{m-1}\frac{\log r_{i}}{r_{i}}\right)$ with equality if and only if the $X_{i}$ (i=1,2,…,m−1)—and hence $Y_{m}$—are i.i.d. normal. The result at order *m* follows by combining the two inequalities since $\left(1-\lambda_{m}\right)s_{m}^{\prime }=r^{\prime }$. ☐

## 5. Recent Versions of the Rényi EPI

**Definition** **5** (Rényi Entropy Power [13]). *The Rényi entropy power of order r is defined by*

(54) $$N_{r}\left(X\right)=e^{2h_{r}\left(X\right)/n}.$$

Up to a multiplicative constant, $N_{r}\left(X\right)$ is the (average) power of a white normal variable having the same Rényi entropy as *X*—hence the name “entropy power”. In fact, if $X^{*}∼N\left(0,\sigma^{2}\right)$ has the same Rényi entropy $h_{r}\left(X^{*}\right)=h_{r}\left(X\right)$, then by Lemma 4,

(55) $$\sigma^{2}=\frac{e^{2h_{r}\left(X\right)/n}}{2\pi r^{r^{\prime }/r}}.$$

The Renyi entropy power enjoys the same scaling property as for the usual power: By Lemma 3, for any a∈R,

(56) $$N_{r}\left(aX\right)=a^{2}N_{r}\left(X\right).$$

For independent $X_{1},X_{2},\ldots ,X_{m}$, Rényi entropy power inequalities take either the form [13,14]

(57) $$N_{r}\left(\sum_{i=1}^{m}X_{i}\right)\geq c\sum_{i=1}^{m}N_{r}\left(X_{i}\right)$$

for some positive constant *c*, or the form [15,16,17]

(58) $${N_{r}^{}}^{\alpha }\left(\sum_{i=1}^{m}X_{i}\right)\geq \sum_{i=1}^{m}{N_{r}^{}}^{\alpha }\left(X_{i}\right)$$

for some positive exponent α. The constants *c* and α may depend on the order *r*, the number *m* of variables and the dimension *n*. What is desired is:

- a maximum possible value of *c* in Equation (57) since the inequality is automatically satisfied for all positive constants $c^{\prime }<c$.
- a minimum possible value of α in Equation (58) since the inequality is automatically satisfied for all positive exponents $\alpha^{\prime }>\alpha$; in fact, since Equation (58) is homogeneous by scaling the variables $X_{i}\mapsto aX_{i}$ as in Equation (56), one may suppose without loss of generality that the r.h.s. of Equation (58) is =1; then, $N_{r}\left(X_{i}\right)<1$, hence Nr2α′(Xi)<Nr2α(Xi) for all *i* and $1\;=\;\sqrt[{\alpha }]{\sum_{i=1}^{m}{N_{r}^{}}^{\alpha }\left(X_{i}\right)}\geq \sqrt[{\alpha^{\prime }}]{\sum_{i=1}^{m}{N_{r}^{}}^{\alpha^{\prime }}\left(X_{i}\right)}$.

The following useful characterization, which generalizes ([16] Lemma 2.1), makes the link between the various versions (Equations (53), (57), and (58)) of the Rényi entropy power inequality.

**Lemma** **8.** *For independent $X_{1},X_{2},\ldots ,X_{m}$, the Rényi EPI in the general form*

(59) $${N_{r}^{}}^{\alpha }\left(\sum_{i=1}^{m}X_{i}\right)\geq c\sum_{i=1}^{m}{N_{r}^{}}^{\alpha }\left(X_{i}\right)$$

*for some constant c>0 and exponent α>0 is equivalent to the following inequality*

(60) $$h_{r}\left(\sum_{i=1}^{m}\sqrt{\lambda_{i}}X_{i}\right)-\sum_{i=1}^{m}\lambda_{i}h_{r}\left(X_{i}\right)\geq \frac{n}{2}\left(\frac{\log c}{\alpha }+\left(\frac{1}{\alpha }-1\right)H\left(\lambda \right)\right)$$

*for any positive $\lambda_{1},\lambda_{2},\ldots ,\lambda_{m}$ such that $\sum_{i=1}^{m}\lambda_{i}=1$, where H(λ)>0 denotes the discrete entropy*

(61) $$H\left(\lambda \right)=\sum_{i=1}^{m}\lambda_{i}\log\frac{1}{\lambda_{i}}>0.$$

**Proof.**  Suppose Equation (59) holds. Then,

(62) $$\begin{array}{cc} h_{r}\left(\sum_{i=1}^{m}\sqrt{\lambda_{i}}X_{i}\right) & =\frac{n}{2\alpha }\log{N_{r}^{}}^{\alpha }\left(\sum_{i=1}^{m}\sqrt{\lambda_{i}}X_{i}\right) \end{array}$$

(63) $$\begin{array}{c} \geq \frac{n}{2\alpha }\log\sum_{i=1}^{m}{N_{r}^{}}^{\alpha }\left(\sqrt{\lambda_{i}}X_{i}\right)+\frac{n}{2\alpha }\log c \end{array}$$

(64) $$\begin{array}{c} =\frac{n}{2\alpha }\log\sum_{i=1}^{m}\lambda_{i}^{\alpha }{N_{r}^{}}^{\alpha }\left(X_{i}\right)+\frac{n}{2\alpha }\log c \end{array}$$

(65) $$\begin{array}{c} \geq \frac{n}{2\alpha }\sum_{i=1}^{m}\lambda_{i}\log\left(\lambda_{i}^{\alpha -1}{N_{r}^{}}^{\alpha }\left(X_{i}\right)\right)+\frac{n}{2\alpha }\log c \end{array}$$

(66) $$\begin{array}{c} =\sum_{i=1}^{m}\lambda_{i}h_{r}\left(X_{i}\right)+\frac{n(\alpha -1)}{2\alpha }\sum_{i=1}^{m}\lambda_{i}\log\lambda_{i}+\frac{n}{2\alpha }\log c \end{array}$$

where the scaling property in Equation (56) is used in Equation (64) and the concavity of the logarithm is used in Equation (65). Conversely, suppose that Equation (60) is satisfied for all $\lambda_{i}>0$ such that $\sum_{i=1}^{m}\lambda_{i}=1$. Set $\lambda_{i}\;=\;{N_{r}^{}}^{\alpha }\left(X_{i}\right)/\sum_{i=1}^{m}{N_{r}^{}}^{\alpha }\left(X_{i}\right)$. Then,

(67) $$\begin{array}{cc} {N_{r}^{}}^{\alpha }\left(\sum_{i=1}^{m}X_{i}\right) & =\exp\frac{2\alpha }{n}h_{r}\left(\sum_{i=1}^{m}\sqrt{\lambda_{i}}\frac{X_{i}}{\sqrt{\lambda_{i}}}\right) \end{array}$$

(68) $$\begin{array}{c} \geq \exp\frac{2\alpha }{n}\sum_{i=1}^{m}\lambda_{i}h_{r}\left(\frac{X_{i}}{\sqrt{\lambda_{i}}}\right)\cdot c\cdot \exp\left(1-\alpha \right)\sum_{i=1}^{m}\lambda_{i}\log\frac{1}{\lambda_{i}} \end{array}$$

(69) =c∏i=1mNr2αXiλiλiα−1λi

(70) $$\begin{array}{c} =c\prod_{i=1}^{m}{\left({N_{r}^{}}^{\alpha }\left(X_{i}\right)\lambda_{i}^{-1}\right)}^{\lambda_{i}} \end{array}$$

(71) $$\begin{array}{c} =c{\left(\sum_{i=1}^{m}{N_{r}^{}}^{\alpha }\left(X_{i}\right)\right)}^{\sum_{i=1}^{m}\lambda_{i}} \end{array}$$

(72) $$\begin{array}{c} =c\sum_{i=1}^{m}{N_{r}^{}}^{\alpha }\left(X_{i}\right).\;☐ \end{array}$$

### 5.1. Rényi Entropy Power Inequalities for Orders >1

From Lemma 8 and Corollary 2, it is easy to recover known Rényi EPIs and obtain new ones for orders r>1. In fact, if r>1, then $r^{\prime }>0$ and all $r_{i}^{\prime }$ are positive and $>r^{\prime }$. Therefore, all $r_{i}$ are <r and by monotonicity (Lemma 5),

(73) $$h_{r_{i}}\left(X_{i}\right)\geq h_{r}\left(X_{i}\right)\;\left(i=1,2,\ldots ,m\right).$$

Plugging this into Equation (53), one obtains

(74) $$h_{r}\left(\sum_{i=1}^{m}\sqrt{\lambda_{i}}X_{i}\right)-\sum_{i=1}^{m}\lambda_{i}h_{r}\left(X_{i}\right)\geq \frac{n}{2}r^{\prime }\left(\frac{\log r}{r}-\sum_{i=1}^{m}\frac{\log r_{i}}{r_{i}}\right)$$

where $\lambda_{i}=r^{\prime }/r_{i}^{\prime }$ for i=1,2,…,m. For future reference, define

(75) $$\begin{array}{cc} A(\lambda ) & =|r^{\prime }|\left(\frac{\log r}{r}-\sum_{i=1}^{m}\frac{\log r_{i}}{r_{i}}\right) \end{array}$$

(76) $$\begin{array}{c} =|r^{\prime }|\left(\sum_{i=1}^{m}\left(1-\frac{\lambda_{i}}{r^{\prime }}\right)\log\left(1-\frac{\lambda_{i}}{r^{\prime }}\right)-\left(1-\frac{1}{r^{\prime }}\right)\log\left(1-\frac{1}{r^{\prime }}\right)\right). \end{array}$$

(The absolute value $|r^{\prime }|$ is needed in the next subsection where $r^{\prime }$ is negative.)

This function is strictly convex in $\lambda =(\lambda_{1},\lambda_{2},\ldots ,\lambda_{m})$ because $x\mapsto \left(1-x/r^{\prime }\right)\log\left(1-x/r^{\prime }\right)$ is strictly convex. Note that A(λ) vanishes in the limiting cases where λ tends to one of the standard unit vectors (1,0,…,0), (0,1,0,…,0), …, (0,0,…,0,1) and since every λ is a convex combination of these vectors and A(λ) is strictly convex, one has A(λ)<0.

**Theorem** **4** (Ram and Sason [14]). *The Rényi EPI (57) holds for r>1 and $c=r^{r^{\prime }/r}{\left(1-\frac{1}{mr^{\prime }}\right)}^{mr^{\prime }-1}$.*

**Proof.**  By Lemma 8 for α=1 we only need to check that the r.h.s. of Equation (74) is greater than $\frac{n}{2}\log c$ for any choice of the $\lambda_{i}$’s, that is, for any choice of exponents $r_{i}$ such that $\sum_{i=1}^{m}\frac{1}{r_{i}^{\prime }}\;=\;\frac{1}{r^{\prime }}$. Thus, Equation (57) will hold for $\log c=\min_{\lambda }A\left(\lambda \right)$. Now, by the log-sum inequality ([4] Theorem 2.7.1),

(77) $$\sum_{i=1}^{m}\frac{1}{r_{i}}\log\frac{1}{r_{i}}\geq \left(\sum_{i=1}^{m}\frac{1}{r_{i}}\right)\log\frac{\sum_{i=1}^{m}\frac{1}{r_{i}}}{m}=\left(m-1/r^{\prime }\right)\log\frac{m-1/r^{\prime }}{m}$$

with equality if and only if all $r_{i}$ are equal, that is, the $\lambda_{i}$ are equal to 1/m. Thus, $\min_{\lambda }A\left(\lambda \right)\;=\;r^{\prime }\left(\frac{\log r}{r}+\left(m-1/r^{\prime }\right)\log\frac{m-1/r^{\prime }}{m}\right)$ which yields $c=r^{r^{\prime }/r}{\left(1-\frac{1}{mr^{\prime }}\right)}^{mr^{\prime }-1}$. ☐

An alternate proof is to argue that A(λ) is convex and symmetrical in $\lambda =(\lambda_{1},\lambda_{2},\ldots ,\lambda_{m})$ and is, therefore, minimized when all $\lambda_{i}$ are equal.

**Remark** **5.** *The above constant c is certainly not optimal since equality in Equation (73) holds if and only if the $X_{i}$ are uniformly distributed (Lemma 5) while equality in Equation (53) holds if and only if the $X_{i}$ are identically normally distributed (Corollary 2). Ram and Sason [14] tightened Equation (57) further using optimization techniques, resulting in a constant that depends on the relative values of the entropy powers themselves.*

**Remark** **6.** *It can be noted that $\log c=r^{\prime }\frac{\log r}{r}+\left(mr^{\prime }-1\right)\log\left(1-\frac{1}{mr^{\prime }}\right)<0$ decreases (and tends to $r^{\prime }\frac{\log r}{r}-1$) as m increases; in fact $\frac{\partial \log c}{\partial m}=r^{\prime }\log\left(1-\frac{1}{mr^{\prime }}\right)+\frac{mr^{\prime }}{r^{\prime }m^{2}}<r^{\prime }\left(-\frac{1}{mr^{\prime }}\right)+\frac{1}{m}=0$. Thus, a universal constant independent of m is obtained by taking*

(78) $$c=\underset{m}{\inf}\;r^{r^{\prime }/r}{\left(1-\frac{1}{mr^{\prime }}\right)}^{mr^{\prime }-1}=r^{r^{\prime }/r}\lim_{m\to \infty }{\left(1-\frac{1}{mr^{\prime }}\right)}^{mr^{\prime }-1}=\frac{r^{r^{\prime }/r}}{e}$$

*This was the constant established by Bobkov and Chistyakov [13].*

**Theorem** **5.** *The Rényi EPI (58) holds for r>1 and $\alpha ={\left(1+r^{\prime }\frac{\log_{2}r}{r}+\left(2r^{\prime }-1\right)\log_{2}\left(1-\frac{1}{2r^{\prime }}\right)\right)}^{-1}$ and this value of α cannot be improved using the method of this paper by making it depend on m.*

**Proof.**  By Lemma 8, for c=1, we only need to check that the r.h.s. of Equation (74) is greater than $\frac{n}{2}\left(1/\alpha -1\right)H\left(\lambda \right)$ for any choice of $\lambda_{i}$s, that is, for any choice of exponents $r_{i}$ such that $\sum_{i=1}^{m}\frac{1}{r_{i}^{\prime }}=\frac{1}{r^{\prime }}$. Thus, Equation (58) will hold for $\frac{1}{\alpha }-1=\min_{\lambda }\frac{A(\lambda )}{H(\lambda )}$. By the proof of the preceding theorem, the numerator is minimized when all $\lambda_{i}$ are equal and this also maximizes the entropy =logm in the denominator. However, one cannot conclude yet since the minimum in the numerator is negative.

A stationary point is easily obtained by the Lagrangian method, which implies that $\frac{\partial }{\partial \lambda_{i}}\frac{A(\lambda )}{H(\lambda )}$ is constant independent of *i*. This gives that $\frac{A(\lambda )}{H(\lambda )}\log\lambda_{i}-\log\left(1-\lambda_{i}/r^{\prime }\right)$ is constant, hence a stationary point is obtained when all $\lambda_{i}$ are equal (to 1/m) and the corresponding value of A(λ)/H(λ) is $\left(r^{\prime }\frac{\log r}{r}+\left(mr^{\prime }-1\right)\log\left(1-\frac{1}{mr^{\prime }}\right)\right)/\log m$.

However, the boundary of the domain of A(λ)/H(λ) is the simplex $\left\{\sum_{i}\lambda_{i}=1,\lambda_{i}\geq 0\right\}$ where on each vertex joining two standard unit vectors, A(λ)/H(λ) has the same expression as for m=2. Li [16] showed—this is also easily proved using [17, Lemma 8]—that, for m=2, the minimum is obtained when λ=(1/2,1/2). The corresponding value of A(λ)/H(λ) is $\left(r^{\prime }\frac{\log r}{r}+\left(2r^{\prime }-1\right)\log\left(1-\frac{1}{2r^{\prime }}\right)\right)/\log2$, which is easily seen to be less than $\left(r^{\prime }\frac{\log r}{r}+\left(mr^{\prime }-1\right)\log\left(1-\frac{1}{mr^{\prime }}\right)\right)/\log m$ for any m≥2.

Therefore, the minimum of A(λ)/H(λ) is attained at the boundary when all $\lambda_{i}$ are zero except two of them equal to 1/2. This gives $\frac{1}{\alpha }-1=\left(r^{\prime }\frac{\log r}{r}+\left(2r^{\prime }-1\right)\log\left(1-\frac{1}{2r^{\prime }}\right)\right)/\log2$. ☐

**Remark** **7.** *The case m=2 yields $\alpha =\frac{r-1}{\left(r+1\right)\log_{2}\left(r+1\right)-r\log_{2}r-2}$ which was found by Li [16] who remarked that this value of α is strictly smaller (better) than the value $\alpha =\frac{r+1}{2}$ obtained by Bobkov and Marsiglietti [15].*

*Interestingly, for m>2, the exponent of Theorem 5 cannot be improved by this method. In fact, in the above proof, it is easily seen that $\left(r^{\prime }\frac{\log r}{r}+\left(mr^{\prime }-1\right)\log\left(1-\frac{1}{mr^{\prime }}\right)\right)/\log m$ is negative and increases toward 0 as m increases. Therefore, the exponent α cannot be decreased (improved) as m increases.*

The above value of α is >1. However, using the same method, it is easy to obtain Rényi EPIs with exponent values α<1. This is given by the following Theorem 6.

**Theorem** **6.** *The Rényi EPI (59) holds for r>1, 0<α<1 with $c={\left(m\;r^{r^{\prime }/r}{\left(1-\frac{1}{mr^{\prime }}\right)}^{mr^{\prime }-1}\right)}^{\alpha }/m$.*

**Proof.**  By Lemma 8 we only need to check that the r.h.s. of Equation (74) is greater than $\frac{n}{2}\left((\log c)/\alpha +(1/\alpha -1)H(\lambda )\right)$, that is, A(λ)≥(logc)/α+(1/α−1)H(λ) for any choice of $\lambda_{i}$s, that is, for any choice of exponents $r_{i}$ such that $\sum_{i=1}^{m}\frac{1}{r_{i}^{\prime }}=\frac{1}{r^{\prime }}$. Thus, for a given 0<α<1, Equation (59) will hold for $\log c=\min_{\lambda }\alpha A\left(\lambda \right)-\left(1-\alpha \right)H\left(\lambda \right)$. From the preceding proofs (since both A(λ) and −H(λ) are convex functions of λ), the minimum is attained when all $\lambda_{i}$s are equal. This gives $\log c=\alpha \left(r^{\prime }\frac{\log r}{r}+\left(mr^{\prime }-1\right)\log\left(1-\frac{1}{mr^{\prime }}\right)\right)-\left(1-\alpha \right)\log m$. ☐

### 5.2. Rényi Entropy Power Inequalities for Orders <1 and Log-Concave Densities

If r<1, then $r^{\prime }<0$ and all $r_{i}^{\prime }$ are negative and $<r^{\prime }$. Therefore, all $r_{i}$ are >r and by monotonicity (Lemma 5), the opposite inequality of Equation (73) holds and the method of the preceding subsection fails. For log-concave densities, however, Equation (73) can be replaced by a similar inequality in the right direction.

**Definition** **6** (Log-Concave Density). *A density f is log-concave if logf is concave in it support, i.e., for all 0<μ<1,*

(79) $$f{\left(x\right)}^{\mu }f{\left(y\right)}^{1-\mu }\leq f\left(\mu x+\left(1-\mu \right)y\right).$$

**Lemma** **9.** *If X has a log-concave density, then $h_{p}\left(pX\right)-ph_{p}\left(X\right)=n\log p+\left(1-p\right)h_{p}\left(X\right)$ is concave in p.*

As noted below in Corollary 3, this is essentially a result obtained by Fradelizi, Madiman and Wang [28]. The following alternate proof uses the transport properties seen in Section 4.

**Proof.**  Define r=λp+(1−λ)q where 0<λ<1. By Lemma 1 there exists two diffeomorphisms T,U such that one can write $pX_{p}=T\left(X^{*}\right)$ and $qX_{q}=U\left(X^{*}\right)$. Then, $X^{*}$ has density

(80) $$\frac{1}{p^{n}}f_{p}\left(\frac{T(x^{*})}{p}\right)|T^{\prime }\left(x^{*}\right)|=\frac{1}{q^{n}}f_{q}\left(\frac{U(x^{*})}{q}\right)|U^{\prime }\left(x^{*}\right)|=\frac{1}{p^{\lambda n}q^{(1-\lambda )n}}f_{p}{\left(\frac{T(x^{*})}{p}\right)}^{\lambda }f_{q}{\left(\frac{U(x^{*})}{q}\right)}^{1-\lambda }|T^{\prime }\left(x^{*}\right){|}^{\lambda }{\left|U^{\prime }\left(x^{*}\right)\right|}^{1-\lambda }.$$

Now, by log-concavity, Equation (79) with μ=λp/r,

(81) $$\begin{array}{cc} f_{p}{\left(\frac{T(x^{*})}{p}\right)}^{\lambda }f_{q}{\left(\frac{U(x^{*})}{q}\right)}^{1-\lambda } & =\frac{1}{{∥f∥}_{p}^{\lambda p}{∥f∥}_{q}^{\left(1-\lambda )q\right)}}f{\left(\frac{T(x^{*})}{p}\right)}^{\lambda p}f{\left(\frac{U(x^{*})}{q}\right)}^{(1-\lambda )q} \end{array}$$

(82) $$\begin{array}{c} \leq \frac{1}{{∥f∥}_{p}^{\lambda p}{∥f∥}_{q}^{\left(1-\lambda )q\right)}}f{\left(\frac{\lambda T\left(x^{*}\right)+\left(1-\lambda \right)U\left(x^{*}\right)}{r}\right)}^{r} \end{array}$$

(83) $$\begin{array}{c} =\frac{{∥f∥}_{r}^{r}}{{∥f∥}_{p}^{\lambda p}{∥f∥}_{q}^{\left(1-\lambda )q\right)}}f_{r}\left(\frac{\lambda T\left(x^{*}\right)+\left(1-\lambda \right)U\left(x^{*}\right)}{r}\right). \end{array}$$

Using the arithmetic-geometric mean inequality in Equation (41) and integrating the density in Equation (80) over $x^{*}\in R^{n}$, one obtains

(84) $${\left(p^{\lambda }q^{1-\lambda }\right)}^{n}{∥f∥}_{p}^{\lambda p}{∥f∥}_{q}^{\left(1-\lambda )q\right)}\leq r^{n}{∥f∥}_{r}^{r}.$$

Taking the logarithm yields the announced concavity. ☐

As a side result, it is interesting to note that we have obtained a simple transportation proof of the following varentropy bound:

**Corollary** **3** (Varentropy Bound [28]). *One has $\mathrm{Var}\log f\left(X_{p}\right)\leq n/p^{2}$, that is, $\mathrm{Var}\log f_{p}\left(X_{p}\right)\leq n$.*

**Proof.**  Since $n\log p+\left(1-p\right)h_{p}\left(X\right)$ is concave, one has $\frac{\partial^{2}}{\partial p^{2}}\left(n\log p+\left(1-p\right)h_{p}\left(X\right)\right)\leq 0$, that is,

(85) $$\frac{\partial^{2}}{\partial p^{2}}\left(\left(1-p\right)h_{p}\left(X\right)\right)\leq \frac{n}{p^{2}}.$$

Differentiating twice using Leibniz’s product rule and plugging the identity in Equation (12), the l.h.s. of this inequality becomes

(86) $$-\frac{1}{1-p}\frac{\partial D(f_{p}∥f)}{\partial p}=\frac{\partial }{\partial p}\int f_{p}\log f=\int f_{p}\log^{2}f-{\left(\int f_{p}\log f\right)}^{2}.\;☐$$

As another easy consequence of Lemma 9, since $n\log p+\left(1-p\right)h_{p}\left(X\right)$ is concave and vanishes for p=1, the slopes $\frac{n\log p+\left(1-p\right)h_{p}\left(X\right)-0}{p-1}$ are non-increasing in *p*. In other words, $h_{p}\left(X\right)+n\frac{\log p}{1-p}$ is nondecreasing. Therefore:

**Corollary** **4** (Marsiglietti and Melbourne [17]). *If p<q then for any X with log-concave density, $h_{p}\left(X\right)\;+\;n\frac{\log p}{1-p}\leq h_{q}\left(X\right)+n\frac{\log q}{1-q}$.*

We can now use Lemma 8 and Corollary 2 to obtain Rényi EPIs for orders r<1. Since all $r_{i}$ are >r, by Corollary 4,

(87) $$h_{r_{i}}\left(X\right)+n\frac{\log r_{i}}{1-r_{i}}\geq h_{r}\left(X\right)+n\frac{\log r}{1-r}\;\left(i=1,2,\ldots ,m\right).$$

Plugging this into Equation (53), one obtains

(88) $$\begin{array}{cc} h_{r}\left(\sum_{i=1}^{m}\sqrt{\lambda_{i}}X_{i}\right)-\sum_{i=1}^{m}\lambda_{i}h_{r}\left(X_{i}\right) & \geq n\left(\frac{\log r}{1-r}-\sum_{i=1}^{m}\lambda_{i}\frac{\log r_{i}}{1-r_{i}}\right)+\frac{n}{2}r^{\prime }\left(\frac{\log r}{r}-\sum_{i=1}^{m}\frac{\log r_{i}}{r_{i}}\right) \end{array}$$

(89) $$\begin{array}{c} =\frac{n}{2}r^{\prime }\left(\sum_{i=1}^{m}\frac{\log r_{i}}{r_{i}}-\frac{\log r}{r}\right) \end{array}$$

where we have used that $\lambda_{i}=r^{\prime }/r_{i}^{\prime }$ for i=1,2,…,m. Notice that the r.h.s. of Equation (89) for r<1 ($r^{\prime }<0$) is the opposite of that of Equation (74) for r>1 ($r^{\prime }>0$). However, since $r^{\prime }$ is now negative, the r.h.s. is exactly equal to $\frac{n}{2}A\left(\lambda \right)$ which is still convex and negative. For this reason, the proofs of the following theorems for r<1 are such repeats of the theorems obtained previously for r>1.

**Theorem** **7.** *The Rényi EPI (57) for log-concave densities holds for $c=r^{-r^{\prime }/r}{\left(1-\frac{1}{mr^{\prime }}\right)}^{1-mr^{\prime }}$ and r<1.*

**Proof.**  It is identical to that of Theorem 4 except for the change $|r^{\prime }|=-r^{\prime }$ in the expression of A(λ). ☐

**Theorem** **8.** *The Rényi EPI (58) for log-concave densities holds for r<1 and $\alpha ={\left(1+|r^{\prime }|\frac{\log_{2}r}{r}+\left(2\right|r^{\prime }\left|+1\right)\log_{2}\left(1+\frac{1}{2|r^{\prime }|}\right)\right)}^{-1}$ and this value of α cannot be improved using the method of this paper by making it depend on m.*

**Proof.**  It is identical to that of Theorem 5 except for the change $|r^{\prime }|=-r^{\prime }$ in the expression of A(λ). ☐

**Remark** **8.** *The case m=2 yields $\alpha =\frac{1-r}{\left(r+1\right)\log_{2}\left(r+1\right)-r\log_{2}r-2r}$ which was found by Marsiglietti and Melbourne [17]. Again, the exponent of the theorem is not improved for m>2.*

**Theorem** **9.** *The Rényi EPI in Equation (59) for log-concave densities holds for $c={\left(mr^{-r^{\prime }/r}{\left(1-\frac{1}{mr^{\prime }}\right)}^{1-mr^{\prime }}\right)}^{\alpha }/m$ where r<1 and 0<α<1.*

**Proof.**  It is identical to that of Theorem 6 except for the change $|r^{\prime }|=-r^{\prime }$ in the expression of A(λ). ☐

## 6. Conclusions

This article provides a comprehensive framework to derive known Rényi entropy power inequalities (with shorter proofs), and prove new ones. The framework is based on a transport argument from normal densities and a change of variable by rotation. Only basic properties of Rényi entropies are used in the proofs.

In particular, the α-modification of the EPI is recovered for two or more independent variables for Rényi entropy orders >1 as well as for orders <1, and the Rényi EPI with multiplicative constant *c* is extended to Rényi entropy orders <1. In addition, a more general formulation with both exponent α and constant *c* is obtained for all orders. In passing, a simple proof using normal transport of a recent sharp varentropy bound was obtained for log-concave densities.

As a perspective, the methods developed in this paper can perhaps be generalized to obtain reverse Rényi entropy power inequalities (see, e.g., the discussion in [16]).
